# Acupuncture-related techniques for prevention of type 2 diabetes mellitus in people with pre-diabetes: Protocol for a systematic review and meta-analysis

**DOI:** 10.1097/MD.0000000000031925

**Published:** 2022-11-11

**Authors:** Haoze Guo, Xingquan Wu, Siyuan Lei, Ying Wang, Weichen Sun, Qingwei Yang, Bailin Song

**Affiliations:** a Department of Acupuncture and Tuina, Changchun University of Chinese Medicine, Changchun, China; b Department of Tuina, Affiliated Hospital of Changchun University of Traditional Chinese Medicine, Changchun, China; c Department of Acupuncture and Tuina, Changchun University of Chinese Medicine, Northeast Asia Research Institute of Traditional Chinese Medicine, Changchun, China.

**Keywords:** acupuncture, prediabetes, protocol, systematic review, type 2 diabetes

## Abstract

In modern society, the incidence of diabetes is increasing year by year. Most individuals pass through a phase of prediabetes before developing full blown diabetes. Acupuncture-related has been widely used in the treatment of diabetes, but there is no systematic review of acupuncture-related on the control of blood glucose in prediabetes. Therefore, this study aims to conduct a meta-analysis of the clinical efficacy of acupuncutre-related therapy in preventing the development of diabetes in patients with prediabetes.

**Methods:**

We will search Web of Science, The Cochrane Library, PubMed, Embase, Chinese Biomedical Literature Database (CBM), China National Knowledge Infrastructure (CNKI), Wan-Fang Database, Chinese Scientific Journal Database (VIP Database) from their respective inception dates to September 1, 2022 to identify potentially eligible studies. We will use the Review Manager 5.4 software provided by the Cochrane Collaborative Network for statistical analysis. We then assessed the quality and risk of the included studies and observed the outcome measures.

**Results:**

This meta-analysis further elucidates the protective effects of acupuncture-related techniques on patients with prediabetes.

**Conclusion:**

The purpose of this meta-analysis was to investigate the effect of acupuncture- related techniques on blood glucose in patients with prediabetes, and to provide more methods for clinical prevention of conversion from prediabetes to diabetes.

## 1. Introduction

Diabetes is a group of metabolic diseases characterized by hyperglycemia resulting from defects in insulin secretion, insulin action, or both. The chronic hyperglycemia of diabetes is associated with long-term damage, dysfunction, and failure of different organs, especially the eyes, kidneys, nerves, heart, and blood vessels.^[1]^ It is predicted that 579 million people will have diabetes in 2030 and the number will increase by 51% (700 million) in 2045.^[2]^ Prediabetes is generally defined as blood glucose levels that are above normal but below the threshold for diabetes, is a risk state that defines a high chance of developing diabetes.^[3]^ Different glycaemic measurements to define the pre-diabetic stage exist, including impaired fasting glucose (IFG), impaired glucose tolerance (IGT) and elevated glycosylated hemoglobin (HbA1c).^[4]^ The global prevalence of prediabetes is expected to increase to 470 million people by 2030.^[5]^ Prediabetes poses several threats; there is increased risk of developing type 2 diabetes mellitus (T2DM), and there are risks inherent to the prediabetes state, including microvascular and macrovascular disease.^[6]^ Hyperglycaemia is a well-described risk factor for all-cause mortality, total number of all-age deaths attributable to high fasting plasma was 6.5 million people in 2017,^[7]^ with T2DM accounting for 1 million deaths.^[8]^ At present, people’s awareness of diabetes and prevention awareness is increasing, but the understanding of prediabetes is not deep enough. Persons diagnosed with pre-diabetes are ideal candidates for diabetes prevention efforts.

At present, in clinical practice, comprehensive lifestyle is used to prevent the development of prediabetes to diabetes, such as healthy diet, regular exercise and so on, metformin is the drug of choice.^[9]^ Several trials have demonstrated significant reductions in the risk of developing diabetes mellitus among individuals with prediabetes after lifestyle or drug-based interventions.^[10]^ As an integral part of traditional Chinese medicine, acupuncture and its related techniques have a good therapeutic effect on controlling blood glucose in patients with prediabetes.^[11,12]^ However, there is no systematic review on the efficacy of acupuncture-related therapy in preventing the development of diabetes in patients with prediabetes. This is the first RCTs meta-analysis to investigate the effect of needle-related techniques on the hypoglycemic effect of prediabetes.

## 2. Methods

This protocol which has been reported is based on the Preferred Reporting Items for Systematic Reviews and Meta-Analyses Protocols guidelines^[13]^ and the corresponding checklist used. This systematic review protocol was registered in the PROSPERO International Registry of Systematic Reviews (ID: CRD42022359654).

### 2.1. Study inclusion criteria

#### 2.1.1. Types of studies.

We will be enrolled in a randomized controlled clinical trials.

#### 2.1.2. Interventions.

All randomized controlled trials comparing acupuncture-related techniques with another anti-diabetic agent, lifestyle interventions, placebo, or no intervention for T2DM prevention in patients with pre-diabetes will be included in this study. The duration of intervention has to be a minimum of 4 weeks.

The treatment group consists of various acupuncture-related therapies, including acupuncture, electroacupuncture, scalp acupuncture, ear acupuncture, warm acupuncture-moxibustion, ear acupoint pressing, moxa and various combination therapies such as moxibustion, acupoint embedding or acupuncture, and medicine combination therapy.

#### 2.1.3. Participants.

Adults (older than 18 years) who have pre-diabetes will be eligible for inclusion. In this study, pre-diabetic state involves separate IFG, separate IGT or both.

#### 2.1.4. Outcomes.

The primary outcome will be the incidence of T2DM in patients with pre-diabetes at baseline. Secondary comes will include fasting blood glucose, 2 hours postprandial blood glucose, HbA1c, IFG, IGT. Safety should also be considered, including the incidence of adverse events such as bleeding, pain, hematoma, and syncope, etc.

### 2.2. Search methods

We will search Web of Science, The Cochrane Library, PubMed, Embase, Chinese Biomedical Literature Database (CBM), China National Knowledge Infrastructure (CNKI), Wan-Fang Database, Chinese Scientific Journal Database (VIP Database) from their respective inception dates to September 1 2022 to identify potentially eligible studies. In addition, we also searched the Chinese Clinical Trial Registry and ClinicalTrials.gov (www.ClinicalTrials.gov/) for in-progress trials with unpublished data. Table 1 presents the PubMed search strategy.

**Table 1:** Search strategy for the PubMed database.

Number	Terms
#1	Type 2 diabetes mellitus (all field)
#2	type 2 diabetes (all field)
#3	T2DM (all field)
#4	#1 OR #2-3
#5	Prediabetes (all field)
#6	pre-diabetes (all field)
#7	Impaired glucose regulation (all field)
#8	#5 OR #6-7
#9	Acupuncture (all field)
#10	Needling (all field)
#11	acupoint (all field)
#12	Acupuncture treatment (all field)
#13	Acupunctue needling (all field)
#14	Scalp acupuncture (all field)
#15	Fire needling (all field)
#16	Ear acupuncture (all field)
#17	Intradermal needling (all field)
#18	Auricular acupuncture (all field)
#19	Electroacupuncture (all field)
#20	Catgut embedding (all field)
#21	Catgut embedding (all field)
#22	#9 OR #10-21
#23	Randomized controlled trial (all field)
#24	Controlled clinical trial (all field)
#25	Randomly (all field)
#26	Randomized (all field)
#27	Random allocation (all field)
#28	Placebo (all field)
#29	Double-blind method (all field)
#30	single-blind method (all field)
#31	Trials (all field)
#32	#23 OR #24-32
#33	#4 And #8 And #22 And #32

### 2.3. Data collections and analysis

#### 2.3.1. Selection of studies.

**Figure 1.**
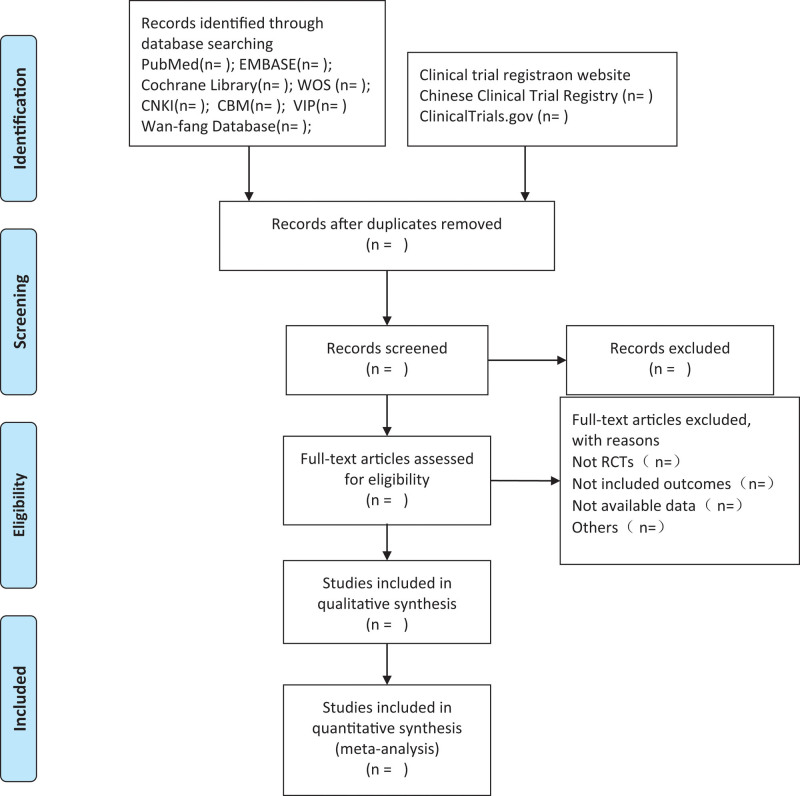
Flow diagram of study selection process.

To ensure that all reviewers have a detailed understanding of the purpose and process of the study, a group meeting will be organized prior to conducting the study. All titles and abstracts were evaluated by 2 reviewers (HG and SL) to exclude irrelevant papers, following specified inclusion criteria. The remaining articles will be included in the further assessment. Reviewers will scrutinize full text for each potentially-relevant article. The inclusion and exclusion process will be described using the Preferred Reporting Items for Systematic Reviews and Meta-Analyses flow diagram (Fig. 1). If there is a disagreement during the review process, it will be reviewed by a third reviewers (WS).

#### 2.3.2. Data extraction and management.

Two reviews (YW and HG) will extract trial information using a standardized data format, and any disagreements arising will be discussed and resolved by a third reviewer (XW). The extracted data is as follows: Participant characteristics (age, sex, ethnicity); methodology (number of participants randomized and analyzed, duration of follow-up); intervention (duration of intervention); HbA1c level (baseline and post-intervention); fasting blood glucose level (baseline and post-intervention); glucose tolerance level (baseline and post-intervention).

### 2.4. Statistical analysis

We will use the Review Manager 5.4 software for statistical analysis. Relative risk was used as the statistical value for dichotomous variable data, for continuous variables, the mean and standard deviation of each study were obtained and pooled as mean difference or standardized mean differences with a 95% confidence interval.

### 2.5. Methodological quality of assessment

This study used the offset risk table proposed by the Cochrane Collaboration Network to assess literature quality. The risk table includes 6 items: random sequence generation mode, whether to use allocation concealment, whether to blind the subjects and intervention providers, whether to blind the results evaluators, whether the results data are complete, whether to select the results report, and other bias sources. The criteria used to assess the risk of bias were “low risk,” “high risk,” and “unclear.” unclear. In this process, 2 evaluators independently evaluated methodological quality.

### 2.6. Assessment of heterogeneity

The assessment of heterogeneity was measure of the χ² test and I² statistic. *P* > .1, *I*^2^ < 50%, considered as low heterogeneity, and fixed effect model was used; *P* < .1, *I*^2^ > 50%, considered as high heterogeneity, random effect model was used. Forest plot and funnel plot are used to show the results.

### 2.7. Assessment of publication bias

If there are >10 trials in accordance with the study, we will use the comparison-adjusted funnel plot to assess small study effects including publication bias at the network level.^[14]^

### 2.8. Grading the quality of evidence

The quality of evidence of estimates derived from this study will be rated using the Grading of Recommendations Assessment, Development and Evaluation (GRADE framework). The GRADE approach characterizes the quality of evidence according to publication bias, study limitations, inconsistency, imprecision, and indirectness, there are 4 levels of results: very low, low, moderate, and high.

### 2.9. Ethics and dissemination

Formal ethical approval was not required for this protocol. Because confidential patient data will not be included in this study, ethical approval is not required for the systematic review.

## 3. Discussion

This study is a comprehensive and systematic review of acupuncture-related therapies comparing various control therapies to prevent the development of type 2 diabetes in patients with prediabetes. Our study will provide a summary of available evidence concerning acupuncture-related agents for T2DM prevention in patients with pre-diabetic state. Acupuncture originated in ancient China and has been widely applied in the clinic for a long time. It is an important component of Eastern medicine, acupuncture-related can effectively improve the blood glucose level of patients, lose weight, promote fat metabolism and enhance the treatment effect.^[15,16]^

We are expecting to discover that, compared with lifestyle interventions, acupuncture-related of TCM can be more helpful for preventing prediabetes eventually developing in to diabetes mellitus.

## Author contributions

**Conceptualization:** Haoze Guo, Xingquan Wu.

**Data curation:** Siyuan Lei.

**Formal analysis:** Ying Wang.

**Funding acquisition:** Bailin Song.

**Investigation:** Weichen Sun.

**Methodology:** Qingwei Yang.

**Supervision:** Bailin Song.

**Validation:** Weichen Sun, Qnigwei Yang.

**Writing – original draft:** Haoze Guo.

**Writing – review & editing:** Haoze Guo, Siyuan Lei.
